# Sulfonated immunoglobulin improves cardiopulmonary functions by promoting IGF-I production in ARDS patients with severe sepsis

**DOI:** 10.1186/cc9838

**Published:** 2011-03-11

**Authors:** Y Deguchi, H Suga, T Sato, N Harada, T Nakagawa, K Okajima

**Affiliations:** 1 Tokyo Women's Medical University MCE, Tokyo, Japan; 2 Nagoya City University Graduate School of Medical Sciences, Aichi, Japan

## Introduction

It has been emphasized that severe sepsis often leads to shock and ARDS in critically ill patients. We reported previously that sulfonated immunoglobulin (sIG) administration significantly inhibited the increase of in lung MPO activities and the increase of pulmonary vascular permeability. In the present study, we examined whether sIG improves not only ARDS but also cardiovascular dysfunction in patients with severe sepsis.

## Methods

ARDS patients with severe sepsis were divided into two groups, the sIG administrated group and the polyethylene glycol-treated immunoglobulin (pIG) administrated group. We evaluated them by measuring the value of IGF-1, lactate, PF ratio, cathecholamine index, septic severity score (SSS) and SOFA score.

## Results

The serum IGF-1 levels in the sIG group were increased at the seventh day significantly (*P *< 0.05). PF ratios in the sIG group were increased significantly at the seventh day (*P *< 0.05). The serum lactate levels and catecholamine index in the sIG group were decreased significantly at the seventh day (*P *< 0.05). The total score of SSS and SOFA also significantly improved in the sIG group at the seventh day (*P *< 0.05).

## Conclusions

These observations suggest that sIG might improve cardiopulmonary functions by promoting IGF-I production in ARDS patients with severe sepsis.
